# Effects of Pterostilbene on the Cell Division Cycle of a Neuroblastoma Cell Line

**DOI:** 10.3390/nu16234152

**Published:** 2024-11-29

**Authors:** Francesca Bruno, Flores Naselli, Desiree Brancato, Sara Volpes, Paola Sofia Cardinale, Salvatore Saccone, Concetta Federico, Fabio Caradonna

**Affiliations:** 1 Department Biological, Geological, and Environmental Sciences, University of Catania, 95124 Catania, Italy; francesca.bruno@unict.it (F.B.); desiree.brancato@phd.unict.it (D.B.); salvatore.saccone@unict.it (S.S.); 2 Department Biological, Chemical and Pharmaceutical Sciences and Technologies, University of Palermo, 90133 Palermo, Italy; flores.naselli@unipa.it (F.N.); sara.volpes@unipa.it (S.V.); paolasofia.cardinale@unipa.it (P.S.C.); fabio.caradonna@unipa.it (F.C.); 3 CERNUT, Interdepartmental Research Center in Nutraceutics and Health Products, 95125 Catania, Italy; 4 NBFC, National Biodiversity Future Center, 90133 Palermo, Italy

**Keywords:** neurodegeneration, cell cycle re-entry, SK-N-BE neuroblastoma cell line, pterostilbene, stilbenoids, nutrigenomic, DNA methylation, tau protein, cyclin *CCND1*

## Abstract

**Background.** The “Cell Cycle Hypothesis” suggests that the abnormal re-entry of neurons into the cell division cycle leads to neurodegeneration, a mechanism supported by in vitro studies on neuronal-like cells treated with the hyperphosphorylating agent forskolin. Pterostilbene, a bioavailable compound found in foods such as blueberries and grapes, may exert neuroprotective effects and could serve as a potential adjunct therapy for neurodegenerative diseases. **Methods**. In this study, we investigated the effects of pterostilbene on neuronal-like cells derived from the human neuroblastoma SK-N-BE cell line, where cell cycle reactivation was induced by forskolin treatment. We analyzed molecular endpoints associated with differentiated versus replicative cell states, specifically the following: (a) the expression of cyclin *CCND1*, (b) the Ki67 cell proliferation marker, (c) the AT8 nuclear tau epitope, and (d) genome-wide DNA methylation changes. **Results**. Our findings indicate that pterostilbene exerts distinct effects on the cell division cycle depending on the cellular state, with neuroprotective benefits observed in differentiated neuronal-like cells, but not in cells undergoing induced division. Additionally, pterostilbene alters DNA methylation patterns. **Conclusion.** These results suggest that pterostilbene may offer neuroprotective advantages for differentiated neuronal-like cells. However, further studies are required to confirm these effects in vivo by examining specific biomarkers in human populations consuming pterostilbene-containing foods.

## 1. Introduction

Aging-related diseases have garnered increasing attention in the scientific community due to the rising global average age. By 2060, it is projected that the elderly will comprise 22% of the total population. Aging is a biological process characterized by a progressive decline in physiological functions, leading to heightened vulnerability to stress, inflammation, and other harmful factors. These changes contribute to the development of various diseases, including neurodegenerative disorders, cardiovascular diseases, malignant tumors, and, ultimately, an increased risk of mortality [1]. For many of these conditions, such as Alzheimer’s disease (AD), the exact molecular mechanisms underlying their onset and progression remain unclear. A key neuropathological hallmark of AD is the presence of Neurofibrillary Tangles (NFTs), which are highly insoluble fibrillar intracellular aggregates composed of hyperphosphorylated tau protein. The accumulation of NFTs is thought to impair neuronal function and contribute to cell death [2,3,4,5].

The “Cell Cycle Hypothesis” is a recent theory proposed to explain the pathogenesis of AD. It suggests that an abnormal re-entry of neurons into the cell division cycle may precede the pathological changes leading to neurodegeneration [6,7,8,9]. Several studies indicate that this cell cycle re-entry might represent an attempt to replace damaged neurons, with the accumulation of Neurofibrillary Tangles (NFTs) potentially reflecting the failure of neurons to repair the damage [10,11,12]. Nucleolar isoforms of the tau protein, such as AT100 (pThr212/Ser214) and AT8 (pSer202/Thr205), have drawn attention for their potential involvement in aging and the early stages of AD. Notably, AT8 displays distinct intracellular localizations depending on the cellular state of the SK-N-BE cell line, distinguishing between replicative (actively dividing) cells and differentiated, neuronal-like (non-dividing) cells [13,14,15]. Furthermore, in neuronal-like cells differentiated from the retinoic acid-induced neuroblastoma cell line SH-SY5Y, forskolin exposure has been shown to induce tau hyperphosphorylation, increase cyclin expression levels, and promote the appearance of mitotic cells [16].

Currently, no definitive therapy exists for age-related neurodegenerative diseases. In this context, the Mediterranean diet has gained attention for its positive effects on cognitive functions and its potential to delay age-related brain decline. These benefits are primarily attributed to its abundance of bioactive compounds, particularly phenolic compounds found in fruits and vegetables, which exhibit significant biological properties [17,18]. A growing body of research is exploring the role of these compounds in reversing or delaying the onset of age-related diseases [19,20]. Stilbenoids, a class of phenolic compounds, are particularly noteworthy for their role in plant defense mechanisms. Their structural core, the stilbene skeleton, is a simple molecule that undergoes various modifications, generating a wide range of derivatives with distinct biological activities [21,22]. Among the stilbenoids, resveratrol (trans-3,5,4′-trihydroxystilbene) and pterostilbene (trans-3,5-dimethoxy-4′-hydroxystilbene) are the most well-known representatives. These compounds have attracted considerable scientific interest due to their potential health-promoting properties, particularly in aging, longevity, and the prevention of age-related diseases [23,24,25,26].

Pterostilbene, found in blueberries, grapes, and grapevines, is a structural analogue of resveratrol and shares many of its properties, including anti-tumor, anti-inflammatory, antioxidant, anti-aging, and anti-obesity effects [24,25]. Its chemical structure enhances lipophilicity, leading to higher bioavailability, improved plasma levels, and greater metabolic stability in the liver, which extends its half-life compared to resveratrol. These characteristics, coupled with reduced toxicity relative to other stilbenoids, make pterostilbene a promising drug candidate for various pathological conditions [27,28,29]. Notably, pterostilbene is recognized for its potential as an epigenetic modulator, as well as for its dose-dependent toxicity and interaction with DNA. In differentiated Caco2 cells, a model of the intestinal epithelial barrier, pterostilbene has demonstrated no toxicity and the ability to reverse DNA demethylation induced by environmental toxins like arsenic. This highlights its potential applications in both preventive and therapeutic contexts [18]. In recent years, various in vitro and in vivo studies have explored the protective effects of pterostilbene in age-related conditions such as dementia and AD [17]. These studies have shown promising results, including the inhibition of inflammatory responses and a reduction in mitochondrial oxidative stress following cerebral ischemic stroke [30]. Furthermore, pterostilbene’s ability to cross the blood–brain barrier enhances its potential as a candidate for modulating brain activity and providing neuroprotective effects [17,31,32,33,34].

Epigenetic alterations, including changes in DNA methylation at CpG sites, play a significant role in neurodegenerative diseases [35,36]. In particular, Parkinson’s Disease (PD) is characterized by global DNA hypermethylation, which appears to contribute to dopamine (DA) depletion, hypokinesia, and tremor observed in both human patients and animal models [37]. However, global hypermethylation in PD can coexist with hypomethylation at specific promoter regions, as recently demonstrated in studies on PD blood samples and cortical tissue [36]. The global hypermethylation observed in PD may be associated with dysfunctions in the enzymatic systems that regulate DNA methylation, such as DNA methyltransferases (DNMTs) or DNA demethylases [38].

In this study, we aimed to evaluate the potential neuroprotective effects of pterostilbene using the human neuroblastoma cell line SK-N-BE, differentiated into neuronal-like cells with retinoic acid. We assessed the effects of pterostilbene on the cell cycle under different physiological conditions by analyzing the expression levels of cyclin *CCND1* and performing the immunodetection of specific markers for replicative cells (Ki67) and differentiated cells (AT8 epitope of tau) in the SK-N-BE cell model [14,16]. Furthermore, building on recent findings that demonstrate pterostilbene’s ability to modulate genome-wide DNA methylation in differentiated human Caco-2 cells [18], we investigated whether similar effects occur in neuronal cell models, potentially offering novel health benefits.

## 2. Materials and Methods

### 2.1. Cell Cultures

The human neuroblastoma cell line SK-N-BE [39] was cultured, under standard conditions of 37 °C and 5% CO_2_, in RPMI 1640 which was supplemented with 10% fetal bovine serum (FBS) and 1% Penicillin/Streptomycin (100 U/mL; 100 μg/mL). The differentiation into neuronal-like cells was obtained by 10 μM retinoic acid (RA) (Sigma-Aldrich, Darmstadt, Germany, Cat. n. R2625) added to the culture medium every 72 h (day 0, 3, 6, 9) for 12 days of treatment [40,41].

Differentiated SK-N-BE cells were treated with 4 μM forskolin (Abcam, Cambridge, UK, Cat. N. AB120058) to induce a restart of the cell cycle, a non-physiological condition that results in the start of cellular degeneration as previously described [16].

SK-N-BE replicative, differentiated by retinoic acid, and differentiated cells induced to neurodegeneration by using Fsk were treated with pterostilbene 10 μM and 100 μM for 4 and 24 h. Cell treatments were performed in culture medium with 1% fetal bovine serum (FBS).

### 2.2. RNA Extraction, and Cyclin Expression Analysis

The RNA extraction from SK-N-BE cells was performed using a MagCore^®^ Compact Automated Nucleic Acid Extractor (RBC Bioscience, New Taipei, Taiwan, Cat. No. MCA0801) in association with the MagCore^®^ Total RNA Cultured Cells Kit (RBC Bioscience, New Taipei, Taiwan, Cat. No. MRC-01). Extracted RNAs were reverse transcribed using the SuperScript III First-Strand Synthesis SuperMix (Invitrogen, Thermo Fisher Scientific, Foster City, CA, USA) to obtain the relative cDNAs.

The quantification of cyclin *CCND1* expression was obtained by qPCR of the cDNAs at the different experimental conditions and was performed by using the StepOne instrument (Applied Biosystems, Foster City, CA, USA). Transcript quantitative detection was conducted with SensiFAST™ SYBR^®^ & Fluorescein Kit (Bioline Reagents, London, UK, Cat. N. BIO-96005) according to the manufacturer’s instructions and experiments were repeated at least three times. The relative quantification method was achieved using actin-b (ACTB, Sigma-Aldrich, Darmstadt, Germany) as an endogenous control. SK-N-BE cells treated with DMSO (vehicle) were used as calibrator references. The 2^−∆∆Ct^ formula was used to evaluate the relative quantification (RQ) of each sample of interest.

### 2.3. Indirect Immunofluorescence Analysis

Indirect immunofluorescence (IIF) experiments were performed on SK-N-BE cells cultured on glass chamber-slides. After treatments, cells were fixed in 4% paraformaldehyde for 20 min at room temperature, and subsequently washed with phosphate-buffered saline (PBS) followed by permeabilization with 0.5% Triton X-100 (Chemsolute, Hamburg, Germany, Cat. N. 8059.0500) as previously described [14]. Immunodetection was performed by overnight incubation at 4 °C with the specific primary antibody. The antibodies were AT8 (Thermo Scientific, Rockford, IL, USA, Cat. N. MN1020; dilution 1:50) to detect phosphorylated pSer202/Thr205 tau and Ki-67 (Invitrogen, Rockford, IL, USA, Cat. N. MA5-14520; dilution 1:100) to detect the cell proliferation marker. Subsequently, cells were incubated for 1 h at 37 °C with FITC-conjugated anti-mouse secondary antibody (Sigma-Aldrich, Saint Louis, MO, USA, Cat. N. F6257; 1:300) and TRITC-conjugated anti-rabbit (Sigma-Aldrich, Saint Louis, Missouri, USA, Cat. N. T6778; 1:400) to perform the dual-color IIF. Cell nuclei were stained with DAPI (blue). Each IIF experiment was repeated at least three times. IIF visualization was performed with a confocal laser scanning microscopy (CLSM) (LSM700, Zeiss, Oberkochen, Baden-Württemberg, Germany) equipped with 40× and 63× objectives. ZEN-2010B SP1 v. 6.0.0.485 software (Zeiss, Oberkochen, Baden-Württemberg, Germany) was used for image acquisition and analysis.

### 2.4. Statistical Analysis

Statistical analyses were conducted using Prism v. 8.0 (GraphPad Software, San Diego, CA, USA). The normality of the variables was assessed using the Kolmogorov–Smirnov test. Differences between groups were determined using the Student’s t-test and statistical significance was defined as follows: *p* < 0.05 (*), *p* < 0.01 (**), and *p* < 0.001 (***).

### 2.5. Genomic DNA Isolation

Isolation of genomic DNA from replicative SK-N-BE cells was carried out using a MagCore^®^ Compact Automated Nucleic Acid Extractor (RBC Bioscience, New Taipei, Taiwan, Cat. No. MCA0801) in association with the MagCore^®^ Cultured Cells DNA Kit (RBC Bioscience, New Taipei, Taiwan, Cat. No. MCC-02). The obtained DNA was quantified by NanoDrop^®^ ND-1000 [42].

### 2.6. Epigenomic Assessment of DNA Methylation

To assess the possible genome-wide changes in DNA methylation, Methylation-Sensitive Arbitrarily-Primed PCR (MeSAP-PCR) was performed as previously described [43]. This is a technique that involves the methylation-sensitive restriction of genomic DNA, coupled with two consecutive PCR reactions to identify altered methylation patterns at different sites with a preference for those rich in GC. This technique, which provides a qualitative and semi-quantitative estimate of DNA methylation levels at the genomic level, allows us to highlight differences in methylation between genomes [44].

## 3. Results

### 3.1. Effect of Pterostilbene on the Cell Cycle by Cyclin CCND1 Expression

To investigate the effect of pterostilbene on the cell cycle, we used the SK-N-BE cell line under three distinct physiological conditions: (a) replicative cells, characterized by an active cell division cycle; (b) differentiated cells, neuronal-like cells induced by retinoic acid treatment, characterized by the absence of an active cell division cycle; and (c) forskolin-treated differentiated cells, neuronal-like cells induced to re-enter the cell cycle. The presence of an active cell division cycle was assessed by monitoring the expression levels of cyclin *CCND1*, a marker associated with the G0-to-G1 checkpoint transition.

**Figure 1:**
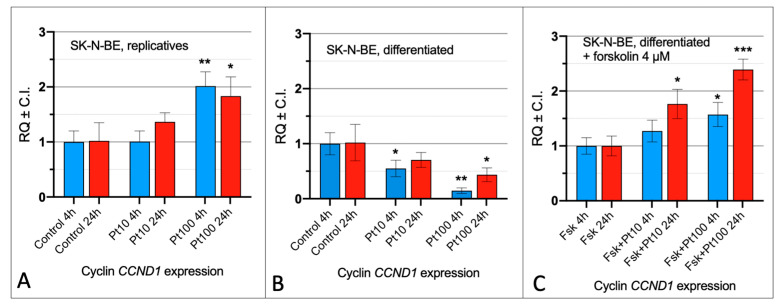
Effect of pterostilbene on the cell cycle of SK-N-BE cells. Expression analysis of cyclin *CCND1* in replicative (**A**), differentiated (**B**), and differentiated with forskolin treatment (**C**). SK-N-BE cells were treated with pterostilbene 10 μM (Pt10) and 100 μM (Pt100) for 4 and 24 h. RQ: relative quantitation obtained by qRT-PCR using the 2^−ΔΔCt^ method. C.I.: Confidence Interval. Control: treatment with DMSO; Fsk: forskolin; Fsk + Pt10: forskolin + pterostilbene 10 µM; Fsk + Pt100: forskolin + pterostilbene 100 µM. *: *p* < 0.05, **: *p* < 0.01, ***: *p* < 0.001.

In the replicative cells, after 4 and 24 h treatments with the lowest concentration of pterostilbene (10 μM), the expression level of cyclin *CCND1* was comparable to the control. However, at the highest concentration (100 μM), a significant increase in cyclin *CCND1* expression was observed (Figure 1A). In differentiated SK-N-BE cells, a dose- and time-dependent decrease in cyclin *CCND1* expression was detected following pterostilbene treatment (Figure 1B). In differentiated cells induced to re-enter the cell cycle with forskolin, a significant increase in cyclin *CCND1* expression was observed with 100 μM pterostilbene treatment at both 4 and 24 h. Conversely, at the lower concentration (10 μM), a significant increase in cyclin *CCND1* expression was observed only after 24 h of pterostilbene treatment (Figure 1C).

### 3.2. Effect of Pterostilbene on the Cell Cycle by Detection of Ki67 and AT8 Markers

To assess the effect of pterostilbene on the cell cycle, we also performed dual-color indirect immunofluorescence (IIF) analysis to examine the presence or absence of the Ki67 proliferative marker and the AT8 epitope of the nuclear tau protein in relation to the physiological states of neuroblastoma cells. This analysis was carried out on SK-N-BE cells treated with pterostilbene under the three physiological conditions described above: replicative, differentiated, and differentiated with forskolin treatment.

**Figure 2:**
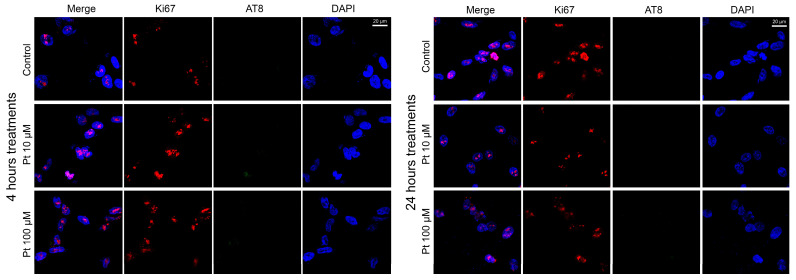
Effect of pterostilbene in SK-N-BE replicative cells. Immunolocalization of Ki67 proliferation marker (red signals) and AT8 tau epitope (green signals) in replicative cells after 4 h (**left panels**) and 24 h (**right panels**) of treatment with pterostilbene. Nuclei were stained with DAPI (blue). Images were captured by means of confocal laser scanning microscope at 400× magnification. Pt: pterostilbene. The scale bar, 20 μm for all the images, is located in the upper right panels.

In the replicative cells treated with 10 μM and 100 μM pterostilbene for 4 and 24 h (Figure 2), we observed the presence of the Ki67 cell proliferation marker in nearly all analyzed cells, along with an almost complete absence of the nuclear AT8 epitope. Specifically, Ki67-positive cells constituted approximately 90% and 94% of the analyzed cells after 4 and 24 h of treatment, respectively. As for the AT8 epitope, it was absent in nearly all cells, being detected in less than 1% of cells at both 4 and 24 h of treatment (Supplementary Figure S1). In this and the following similar analyses, we evaluated the presence or absence of the IIF signal for Ki67 and AT8 above the threshold level. The intensity of the fluorescent signal was not analyzed, as our aim was to determine the number of cells with Ki67 or AT8, independently of signal intensity, to assess whether the cell cycle had started or arrested.

**Figure 3:**
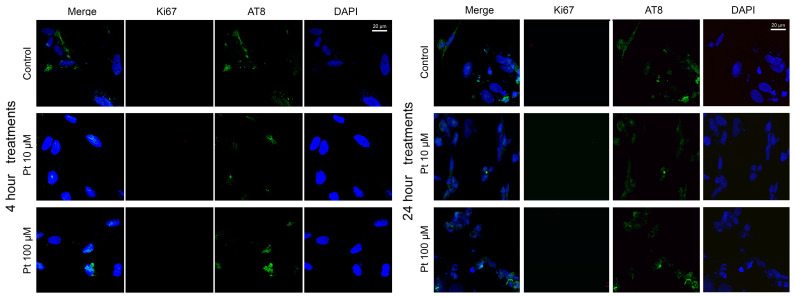
Effect of pterostilbene in differentiated SK-N-BE cells. Immunolocalization of Ki67 proliferation marker (red signals) and AT8 tau epitope (green signals) in differentiated SK-N-BE cells after 4 h (**left panels**) and 24 h (**right panels**) treatment with pterostilbene. Nuclei were stained with DAPI (blue). Images were captured by means of confocal laser scanning microscope at 400× magnification. Pt: pterostilbene. The scale bar, 20 μm for all the images, is located in the upper right panels.

In differentiated cells, treatment with pterostilbene for 4 and 24 h (Figure 3) resulted in an almost complete absence of the Ki67 marker, with less than 2% of cells exhibiting Ki67 expression at both time points (Supplementary Figure S1). This indicates that pterostilbene did not alter the differentiated status of the cells, as further supported by the observed decrease in cyclin *CCND1* expression (Figure 1B). Additionally, the AT8 epitope was predominantly observed in the cytoplasm and nucleolus of a large proportion of cells (Figure 3), with approximately 90% of cells showing AT8-positive cells at both 4 and 24 h of treatment. No significant changes in the percentage of AT8-positive cells were detected at 10 μM and 100 μM concentrations of pterostilbene compared to untreated controls (Supplementary Figure S1).

**Figure 4:**
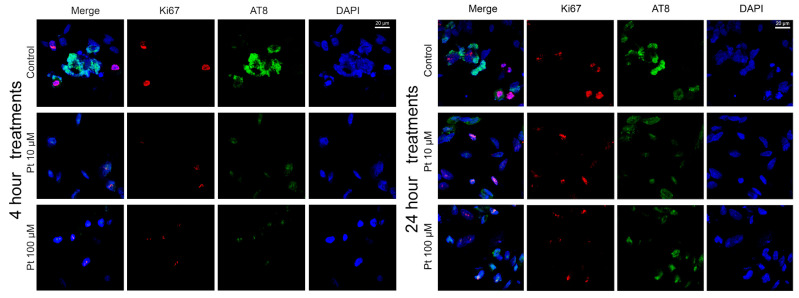
Effect of pterostilbene in SK-N-BE cells induced to cell cycle re-activation by forskolin. Immunolocalization of Ki67 proliferation marker (red signals) and AT8 tau epitope (green signals) in forskolin induced cells after 4 h (**left panels**) and 24 h (**right panels**) of pterostilbene treatment. Nuclei were stained with DAPI (blue). Images were captured by means of confocal laser scanning microscope at 400× magnification. Pt: pterostilbene. The scale bar, 20 μm for all the images, is located in the upper right panels.

Cell cycle reactivation in differentiated neuronal-like SK-N-BE cells was induced using forskolin, which triggers tau hyperphosphorylation—a hallmark commonly associated with the early stages of neuronal degeneration. To assess whether pterostilbene could reverse or inhibit forskolin-induced cell cycle reactivation, we analyzed the presence of the Ki67 proliferation marker as an indicator of cell cycle activity, along with the AT8 epitope, which is associated with cell differentiation. In differentiated SK-N-BE cells treated with both forskolin and pterostilbene, no significant changes in the expression of Ki67 or the AT8 epitope were observed compared to control cells at either of the tested pterostilbene doses after 4 or 24 h of treatment (Figure 4). Specifically, Ki67 was detected in approximately 24% and 27% of cells after 4 and 24 h, respectively, while the AT8 epitope was observed in 73% and 69% of cells at the same time points (Supplementary Figure S1).

### 3.3. Effects of Pterostilbene on the Genome-Wide DNA Methylation Pattern

To analyze the alteration of the DNA methylation pattern following treatments with pterostilbene at both concentrations of 10 μM and 100 μM at two time points (4 h and 24 h), we performed the MeSAP PCR using the experimental models described in the Section 2.

**Figure 5:**
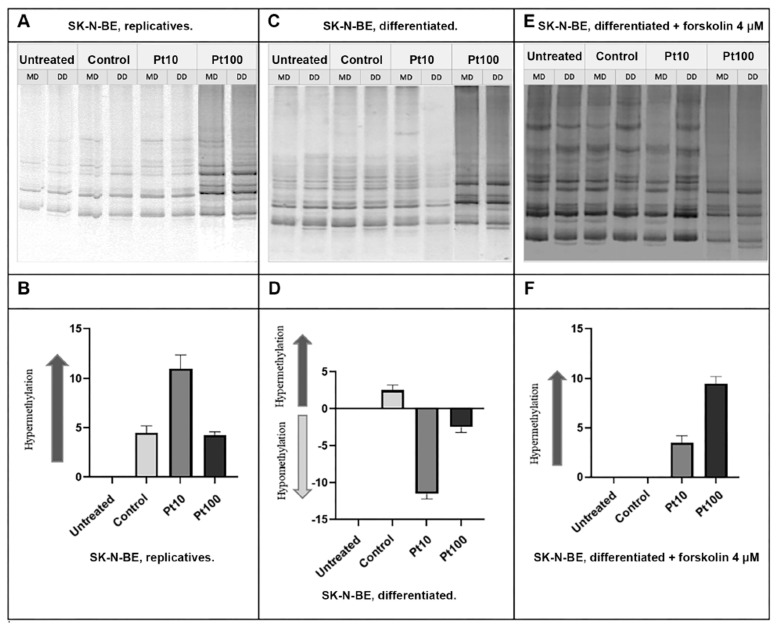
DNA methylation pattern on the SK-N-BE cells after 4 h of pterostilbene treatment. Representative MeSAP-PCR fingerprinting of replicative (**A**), differentiated (**C**), and forskolin-treated SK-N-BE cells (**E**) and the related graphic representations of the densitometry analysis (**B**, **D**, and **F** respectively). Control: cells treated with DMSO; Pt10 and P100: cells treated with pterostilbene 10 μM and 100 μM, respectively. Band pattern variation, in terms of intensification/weakening and appearance/disappearance, was evaluated by the densitometer scanning of mono-digested DNA (MD) in comparison with double-digested DNA (DD).

The results obtained after 4 h of treatment showed significant changes in DNA methylation patterns (Figure 5). In particular, treatment with both pterostilbene 10 μM and 100 μM on replicative cells induced hypermethylation. Contrarily, in differentiated cells, treatments with both pterostilbene 10 μM and 100 μM led to global hypomethylation. In forskolin-treated cells, both pterostilbene 10 μM and 100 μM caused the hypermethylation of the global DNA methylation pattern, similarly to replicative cells.

**Figure 6:**
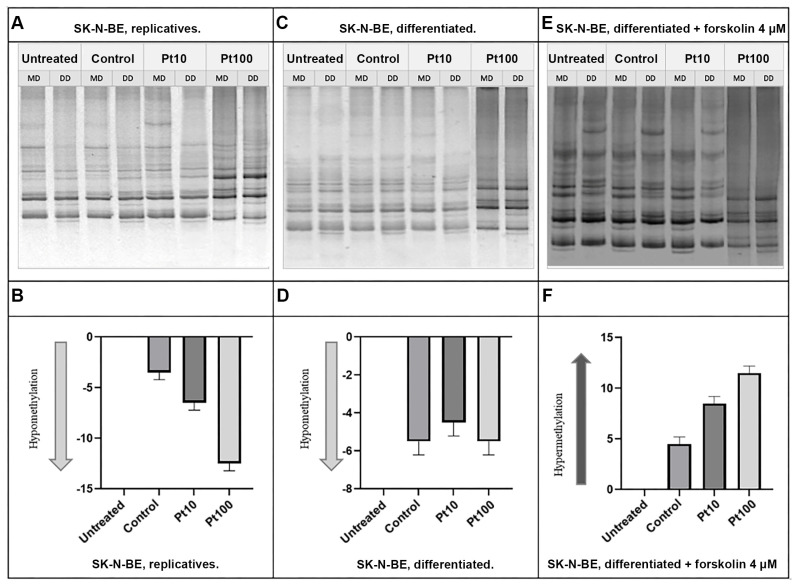
DNA methylation pattern on the SK-N-BE cells after 24 h of pterostilbene treatment. Representative MeSAP-PCR fingerprinting of replicative (**A**), differentiated (**C**), and forskolin-treated SK-N-BE cells (**E**) and the related graphic representations of the densitometry analysis (**B**, **D**, and **F** respectively). Control: cells treated with DMSO; Pt10 and P100: cells treated with pterostilbene 10 μM and 100 μM, respectively. Band pattern variation, in terms of intensification/weakening and appearance/disappearance, was evaluated by the densitometer scanning of mono-digested DNA (MD) in comparison with double-digested DNA (DD).

After 24 h of treatment (Figure 6), pterostilbene at both concentrations, 10 μM and 100 μM, induced hypomethylation in the DNA methylation patterns of replicative cells. However, in differentiated cells, prolonged treatments to 24 h did not result in any significant changes in the DNA methylation pattern. In contrast, in forskolin-treated cells, treatments with both pterostilbene 10 μM and 100 μM led to hypermethylation, similar to the results observed after 4 h of treatment.

## 4. Discussion

Currently, treatments for Alzheimer’s Disease (AD) and other neurodegenerative diseases only alleviate symptoms and do not halt or reverse disease progression [45]. Therefore, identifying the early events of neurodegeneration (ND) that typically occur at the onset of AD, along with discovering nutraceutical compounds with nutrigenomic activity capable of preventing or counteracting ND in its initial stages, represents a goal of significant scientific and clinical impact. Some studies have reported an inverse association between cancer and AD, suggesting that the mechanisms initiating AD pathogenesis might protect against cancer, and vice versa [46]. This implies that AD and cancer could be linked through shared molecular mechanisms related to aging, particularly involving structural changes in nuclear chromatin [47,48], potentially driven by epigenetic modifications. Given that one of the primary nutrigenomic effects of dietary molecules is their influence on genome-wide DNA methylation [49], we analyzed specific molecular endpoints in an appropriate in vitro cell system, as a preliminary model previously used for screening the molecular targets of various exogenous compounds [50,51]. Our aim was to explore potential links between neurodegeneration and the protective effects of dietary molecules typical of the Mediterranean diet, with the ambitious goal of identifying food-derived compounds that could combat neurodegeneration in human cells.

We selected pterostilbene, a bioavailable stilbenoid found in grapes and grapevines, and a key component of the Mediterranean diet, to investigate its potential neuroprotective effects. Our analysis focused on three molecular endpoints: (i) expression of the cell cycle proliferation markers cyclin *CCND1* and Ki67 [2,52]; (ii) expression of the AT8 epitope, a marker of neuronal differentiation related with nuclear tau protein [14]; and (iii) genome-wide DNA methylation changes [18].

To achieve this, we utilized an in vitro cell system derived from the SK-N-BE neuroblastoma cell line under three distinct cellular conditions: (i) replicative, (ii) differentiated, and (iii) differentiated cells induced to re-enter the cell division cycle with forskolin. While it is true that the cell line used in this research differs from typical human neurons, as it is a tumor cell line, we believe its advantages outweigh its limitations. Specifically, the selection of a neuroblastoma cell line was driven by its ability to differentiate into neuron-like cells upon retinoic acid treatment and to re-enter the cell division cycle when stimulated with molecules like forskolin. This makes it a highly valuable model for studying neurotoxicity and assessing the potential anti-neurodegenerative effects of various compounds, including natural substances such as pterostilbene. Additionally, the use of neuroblastoma cells as an in vitro model is well supported by numerous prior studies on neurodegenerative diseases. Their capacity to survive in culture for extended periods enables prolonged experiments with high reproducibility, thereby minimizing experimental variability.

This approach was based on the cell cycle hypothesis underlying neuronal cell degeneration, as well as the role of nuclear tau protein—specifically the AT8 epitope—as a significant marker of neuronal differentiation in neuroblastoma cell lines, as previously described [14,16]. Therefore, this molecular endpoint is particularly valuable as a marker for events occurring in the early stages of AD. When used alongside markers indicative of active cell cycle progression, such as Ki67 or cyclin *CCND1* expression, it provides a comprehensive framework for studying the molecular mechanisms of neurodegeneration.

The number of differentiated neuroblastoma cells treated with pterostilbene positive for Ki67 and AT8 did not show statistically significant variations. However, cyclin *CCND1* exhibited a statistically significant decrease at the highest concentration and longest exposure time, suggesting a contrasting action against the activation of an ectopic cell cycle. This effect, however, was not observed in replicative cells or those induced to re-enter the cell division cycle by forskolin. Indeed, the expression level of cyclin *CCND1*, a marker specific to the transition from the G0 to G1 phase of the cell cycle, revealed contrasting effects of pterostilbene in SK-N-BE cells depending on their cell division status. In differentiated cells, pterostilbene exhibited an inhibitory effect on cyclin *CCND1* expression, observable even after just 4 h of treatment at the lowest concentration tested. Conversely, in replicative cells, pterostilbene induced an increase in cyclin *CCND1* expression, though only at the highest dose tested (100 μM). A similar effect was observed in differentiated cells treated with forskolin, suggesting that pterostilbene affects these cells in a manner similar to replicative cells. Thus, pterostilbene is unable to counteract the reactivation of the cell cycle; in fact, in differentiated cells treated with forskolin, its effect does not reduce the proportion of replicative cells expressing Ki67, nor does it significantly alter the proportion of cells expressing AT8, which remains almost constant.

These findings suggest a potential protective role for pterostilbene in differentiated cells, enabling them to maintain their differentiated state by significantly reducing cyclin *CCND1* expression and thereby decreasing the likelihood of ectopic cell cycle reactivation, even if this is not supported by the proportion of Ki67-positive cells. In contrast, in cells undergoing active division, pterostilbene appears to promote cell proliferation. Consequently, while pterostilbene may not be beneficial once neurodegeneration has commenced, by means of an ectopic restart of the cell division cycle, its neuroprotective effects in healthy neurons are promising, even at low doses.

Growing evidence supports the involvement of aberrant DNA methylation in the disruption of key processes such as the development, proliferation, differentiation, and maintenance of oligodendrocyte cells, which are often impaired in neurodegenerative diseases [53]. Thus, assessing genome-wide DNA methylation in untreated, treated, and co-treated cells is a modern and valuable endpoint, especially when combined with treatments that alter neuronal cell cycle progression and viability. By evaluating genome-wide DNA methylation, we gained insights into the epigenomic potential of natural food-derived compounds in relation to neuronal cell cycle progression, failure, and neurodegeneration. Recent research has also highlighted the role of mitochondrial DNA (mtDNA) methylation as a regulator of various cellular functions. However, mtDNA methylation primarily occurs at non-CpG sites, rather than CpG sites [54]. In this work, for DNA methylation evaluation, the MeSAP-PCR reaction was used. This technique is representative of the entire genome methylation status, targeting preferentially CpG islands, as the used primer is characterized by a “CG tail” [55].

So, given that our technique targets CpG islands, we conclude that mitochondrial DNA methylation contributes minimally to our findings, which are predominantly reflective of genomic DNA methylation. Our previous studies demonstrated that pterostilbene modulates genome-wide DNA methylation in human differentiated Caco-2 cells [18]. In the current research, we used similar concentrations of pterostilbene (and their lower multiples) to treat neuronal cells for 4 or 24 h. These time points were chosen to mimic different stages of DNA methylation dynamics. In fact, during interphase, before DNA replication, de novo DNA methylation occurs, whereas after a full cell cycle, maintenance DNA methylation also takes place.

The results revealed significant changes in DNA methylation patterns depending on the treatment and cell state. In replicative cells, a 4 h treatment with 10 μM pterostilbene induced global DNA hypermethylation. In contrast, in differentiated cells, treatments with both 10 μM and 100 μM pterostilbene for 4 h resulted in global hypomethylation. In forskolin-treated cells, pterostilbene at both 10 μM and 100 μM concentrations caused the global hypermethylation of DNA, resembling the effects observed in replicative cells. After 24 h of treatment, pterostilbene at both concentrations induced global hypomethylation in replicative cells. However, in differentiated cells, prolonged treatment for 24 h did not produce any significant changes in DNA methylation patterns. In contrast, forskolin-treated cells exposed to pterostilbene at both 10 μM and 100 μM concentrations for 24 h exhibited global DNA hypermethylation, consistent with the effects observed after 4 h of treatment.

## 5. Conclusions

The link between neurodegeneration (ND) and genome-wide DNA methylation is well established [38], although the precise mechanisms involved remain unclear. Furthermore, studies on the “cell cycle hypothesis” have underscored the pivotal role of DNA methylation, particularly in cellular dysfunction associated with ND. Our findings suggest that pterostilbene exerts effects on the cell division cycle and supports the hypothesis that neurodegenerative processes are associated with alterations in DNA methylation, particularly hypermethylation. This aligns with the results of Fernández-Santiago et al. (2019) [38], who reported global DNA hypermethylation in dopaminergic neurons from patients with PD. In addition, numerous studies have already demonstrated that alterations in genome-wide DNA methylation are involved in regulating neuroblastoma cell differentiation, leading to functional consequences [50].

Our findings align with evidence that PD-associated hypermethylation can specifically target genes involved in neural functions [56]. The differentially methylated CpG sites were predominantly enriched in intergenic regions. These non-coding regions may contribute to the pathogenesis of human diseases by influencing transcriptional regulatory elements or non-coding RNAs, such as lncRNAs, miRNAs, siRNAs, piRNAs, and snoRNAs. Such alterations could, in turn, affect the regulation of gene expression in other genes, including those involved in the cell cycle, such as cyclin *CCND1* and the MKI67 gene [38].

Several intriguing properties of pterostilbene have been reported, sparking interest in its potential use within a personalized nutrition approach to complement conventional pharmacological treatments for neurodegenerative diseases. In particular, pterostilbene has the following characteristics: (i) it is rapidly absorbed and widely distributed in tissues, (ii) it can easily cross the blood–brain barrier [27], (iii) it has approximately 80% bioavailability [29], and (iv) it exhibits greater metabolic stability compared to other stilbenoids [57].

In conclusion, our findings contribute to the growing body of knowledge on pterostilbene, highlighting its ability to regulate the cell division cycle and induce beneficial changes in DNA methylation. We believe that this stilbenoid shows promise as a potential therapeutic agent for delaying the onset or progression of neurodegenerative diseases, and such research could provide a stronger foundation for developing personalized nutritional strategies aimed at improving patient health outcomes. However, further research is needed to elucidate its precise mechanisms and roles in neurodegenerative diseases. Considering that the present results were obtained using a neuroblastoma cell line, future studies should focus not only on primary neuronal cells but also on in vivo systems.

## Data Availability

All data are contained within the article and in Supplementary Materials.

## Supplementary Materials

The following supporting information can be downloaded at: https://www.mdpi.com/article/10.3390/nu16234152/s1. Original gels for Figure 5 and Figure 6. Figure S1. Statistical analysis of the IIF results.
