# Supplementary material for: Effects of Pterostilbene on the Cell Division Cycle of a Neuroblastoma Cell Line
Source: Nutrients. 2024 Nov 29;16(23):4152. doi: 10.3390/nu16234152 (PMC11644761; doi:10.3390/nu16234152)

Replicative SK-N-BE, 4 and 24 hours

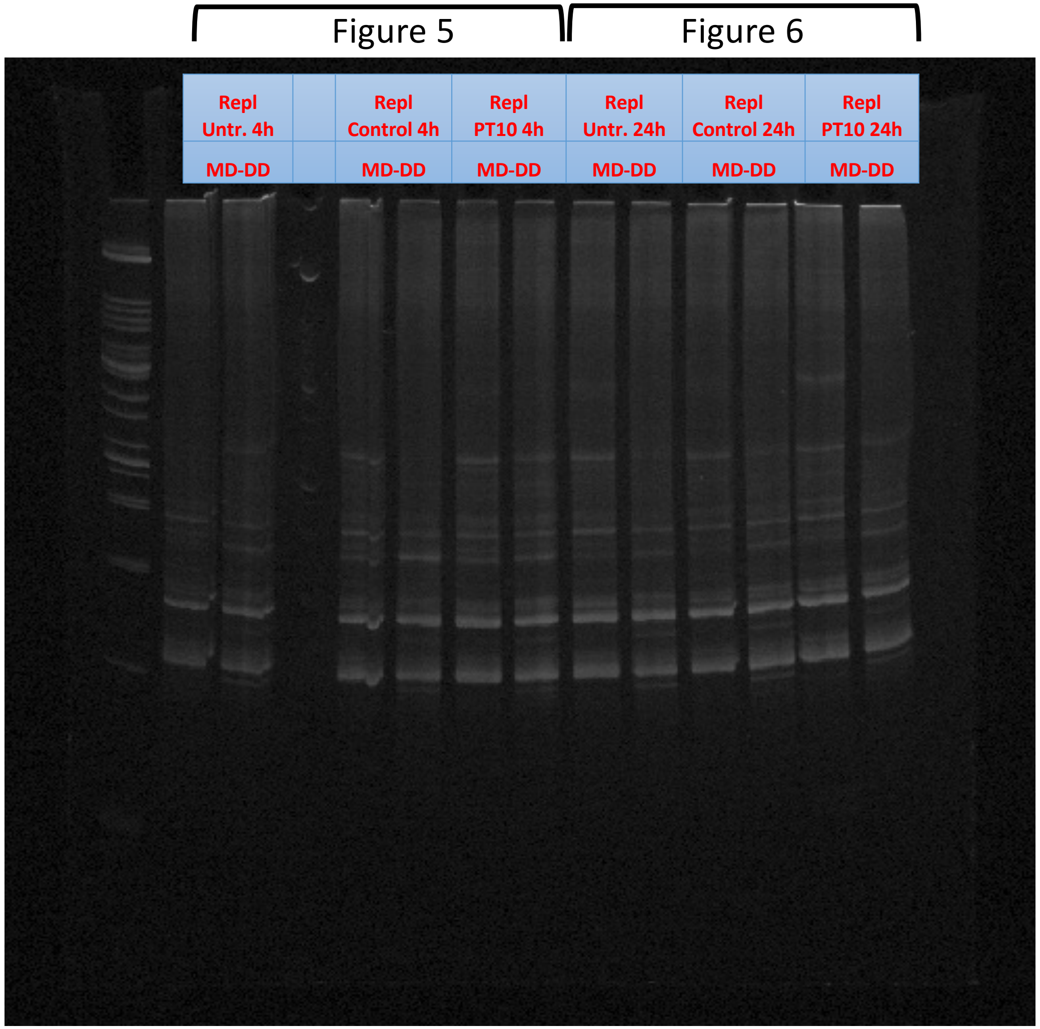

Differentiated SK-N-BE, 4 and 24 hours

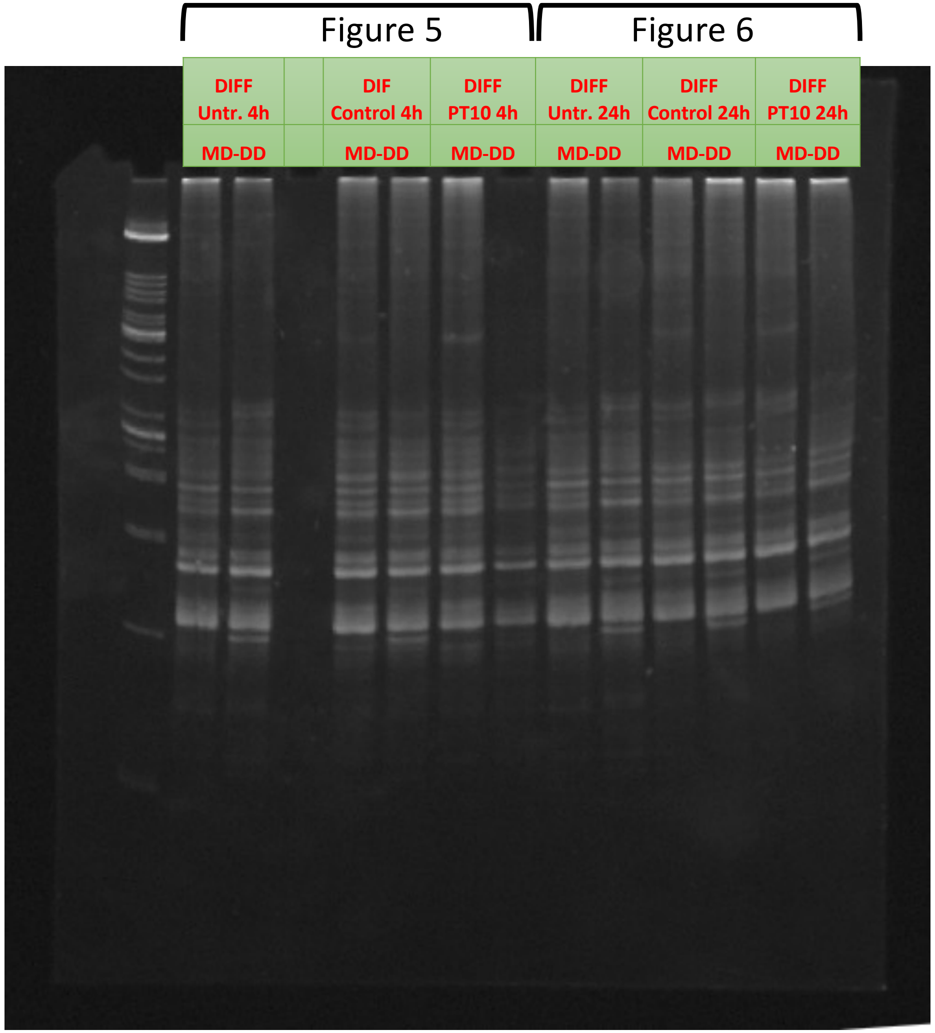

Differentiated SK-N-BE + forskolin, 4 and 24 hours

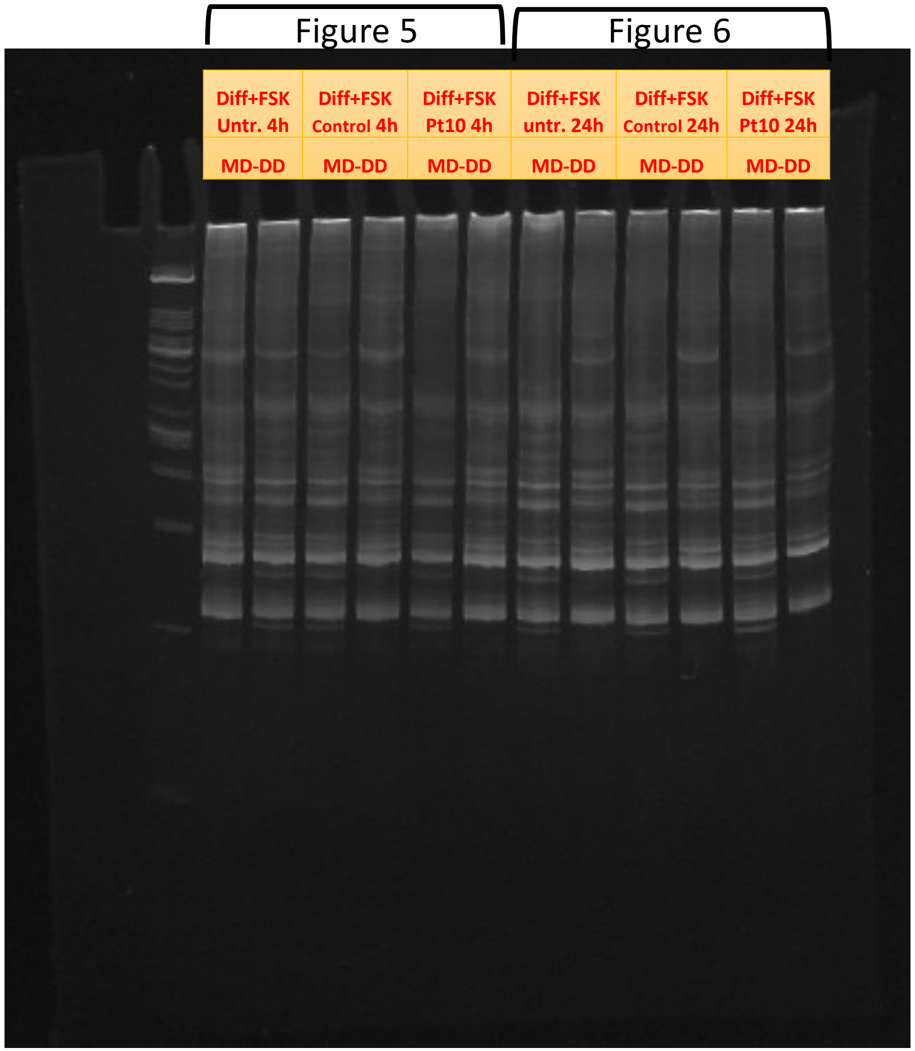

Pterostilbene treatments, 100 µm

Fig.5 Fig. 6. Fig.5 Fig. 6. Fig.5 Fig. 6

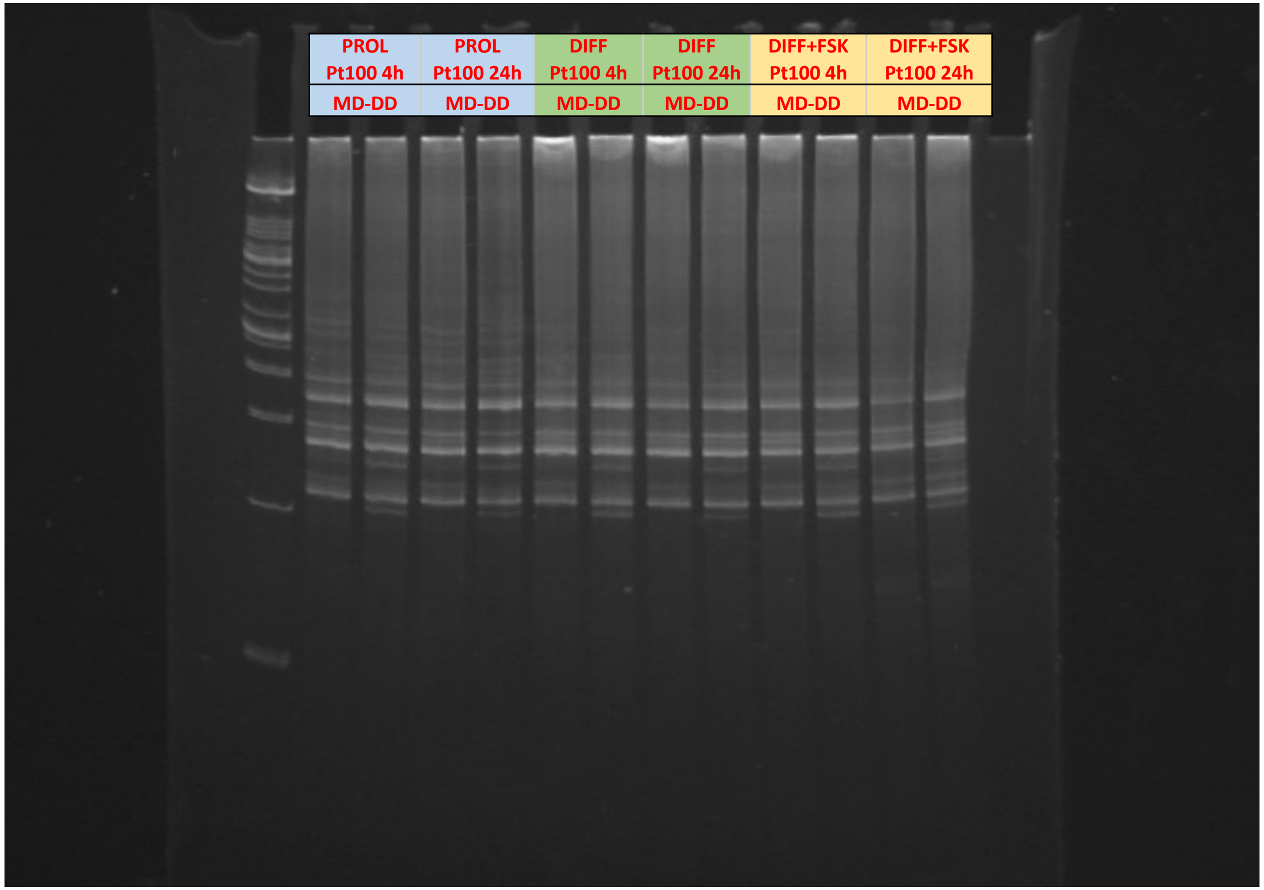

Supplement: Supplementary file 1 [file nutrients-16-04152-s001.zip › Original images of gels/5 correspondence between figures and gels.pdf]
