# Supplementary material for: Effects of Pterostilbene on the Cell Division Cycle of a Neuroblastoma Cell Line
Source: Nutrients. 2024 Nov 29;16(23):4152. doi: 10.3390/nu16234152 (PMC11644761; doi:10.3390/nu16234152)
Supplement: Supplementary file 1 [file nutrients-16-04152-s001.zip › Supplementary Figure S1.pdf]

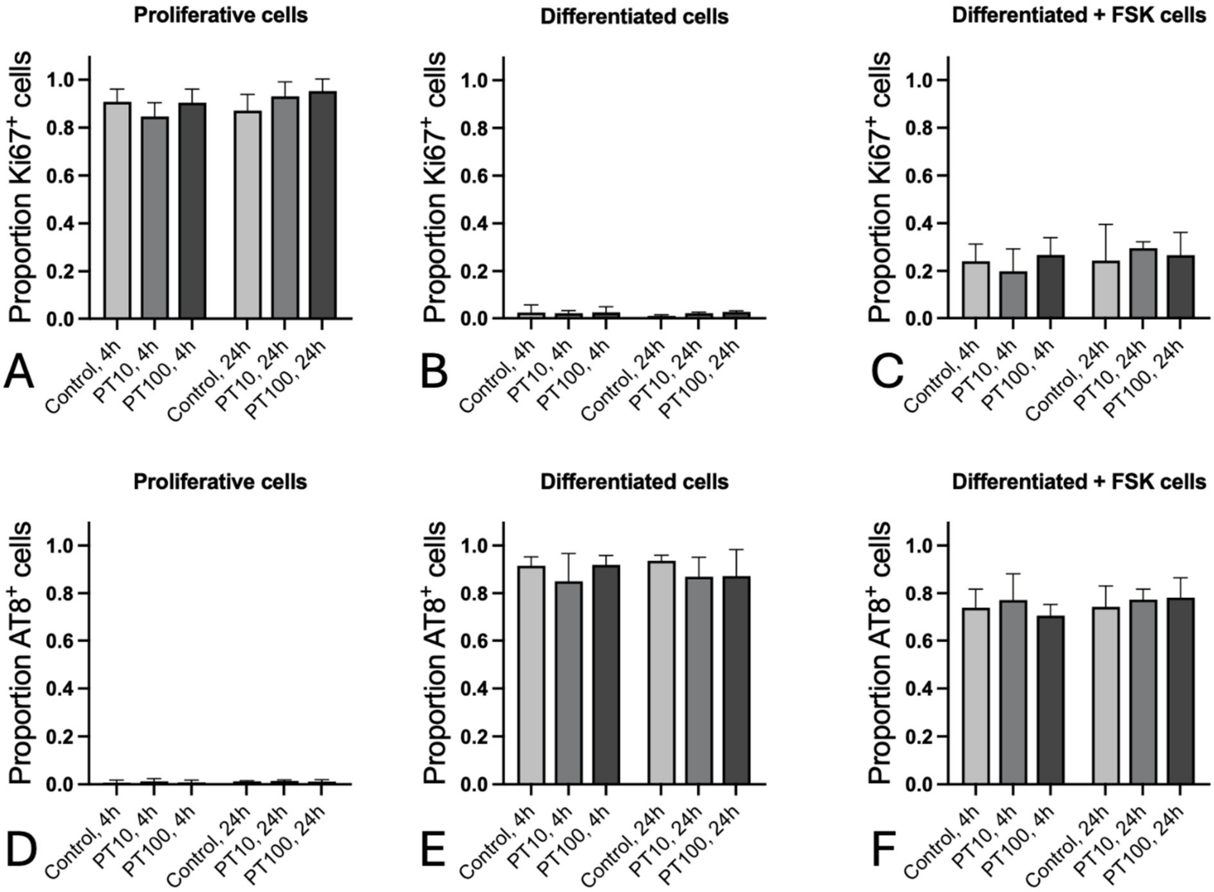

Supplementary Figure S1. Statistical analysis of the IIF results. A, B, and C: proportion of cells showing Ki67 marker in the three different SK-N-BE treatments: proliferative, differentiated and differentiated + forskolin, respectively (see Figs. 2, 3, and 4, respectively). D, E, and F: proportion of cells showing Ki67 marker in the three different SK-N-BE treatments: proliferative, differentiated and differentiated + forskolin, respectively (see Figs. 2, 3, and 4, respectively). PT10, and PT100 indicate treatments with pterostilbene 10 μM, and 100 μM, respectively. Each data represents the mean value ± SD of three tests.
