# Supplementary figures and images for: Effects of Pterostilbene on the Cell Division Cycle of a Neuroblastoma Cell Line
Source: Nutrients. 2024 Nov 29;16(23):4152. doi: 10.3390/nu16234152 (PMC11644761; doi:10.3390/nu16234152)

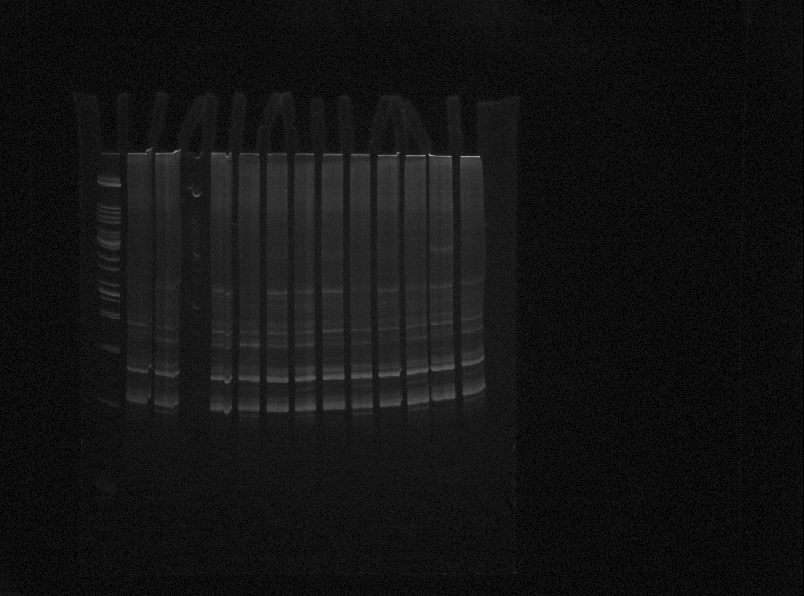

Supplement: Supplementary file 1 [file nutrients-16-04152-s001.zip › Original images of gels/1 MESAP Repl 4-24h.jpg]

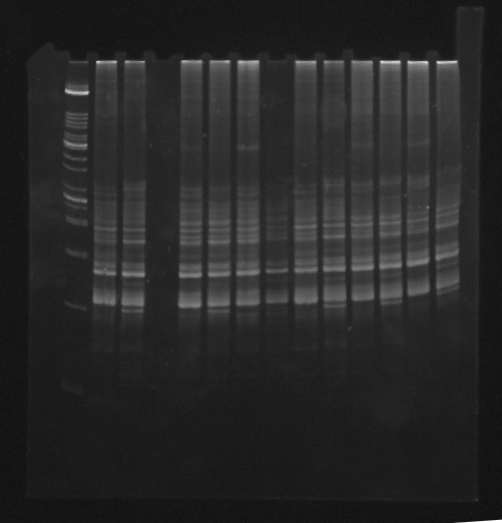

Supplement: Supplementary file 1 [file nutrients-16-04152-s001.zip › Original images of gels/2 MESAP DIFF 4-24h.png]

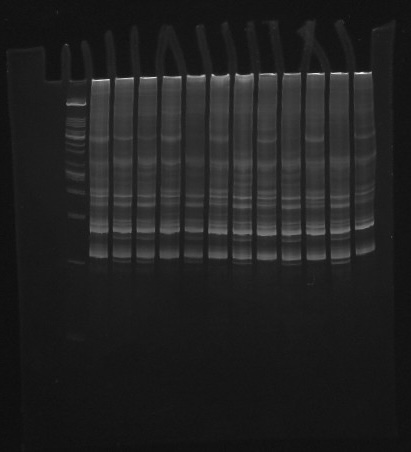

Supplement: Supplementary file 1 [file nutrients-16-04152-s001.zip › Original images of gels/3 MESAP DIFF+FSK 4-24h.jpg]

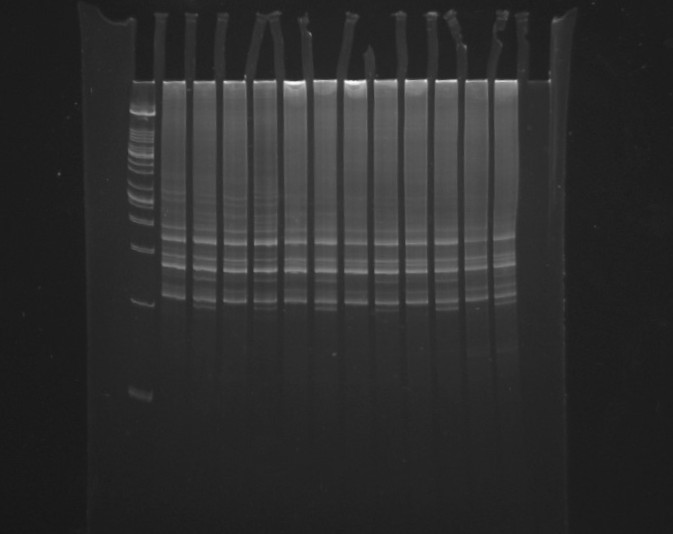

Supplement: Supplementary file 1 [file nutrients-16-04152-s001.zip › Original images of gels/4 MESAP Pt100.jpg]
